# Using period analysis to timely provide serial data on long-term survival for liver cancer patients from China

**DOI:** 10.3389/fonc.2026.1801386

**Published:** 2026-04-14

**Authors:** Pengtao Chen, Wei Gu, Xin Bing, Liangyou Wang, Yongran Cheng, Xukai Chen, Dinghu Zhang, Tianhui Chen

**Affiliations:** 1 Postgraduate Training Base Alliance of Wenzhou Medical University (Zhejiang Cancer Hospital), Wenzhou, China; 2 Department of Cancer Prevention, Zhejiang Cancer Hospital, Hangzhou, China; 3 Hangzhou Institute of Medicine (HIM), Chinese Academy of Sciences, Hangzhou, China; 4 Primary Health Care Department, Wuwei Maternal and Child Health Hospital, Wuwei, China; 5 Department of Non-communicable Chronic Disease Control and Prevention, Taizhou Center for Disease Control and Prevention, Taizhou, China; 6 School of Public Health, Hangzhou Medical College, Hangzhou, China; 7 Department of Interventional Radiology, Zhejiang Cancer Hospital, Hangzhou, China

**Keywords:** cancer screening, liver cancer, long-term survival, period analysis, relative survival

## Abstract

**Background:**

Timely evaluation of long-term survival in liver cancer is essential for assessing early detection and screening efficacy. However, population-based survival data in China remain scarce, particularly for multi-decade analyses. This study systematically evaluated age-standardized 5-year relative survival (RS) in Taizhou, eastern China, and quantified temporal improvements from 2004 to 2023.

**Methods:**

A retrospective cohort of 12,032 liver cancer patients diagnosed in Taizhou (2004–2023) was analyzed. Period analysis was used to compute age-standardized 5-, 10-, 15-, and 20-year RS rates, stratified by gender, age, and urban versus rural residence. Survival estimates were derived using actuarial methods with adjustments for competing mortality risks based on regional life tables.

**Results:**

The 5-year RS for patients during 2019–2023 was 37.32%, significantly exceeding earlier periods (29.13% for 2014-2023, 23.00% for 2009-2023, and 5.56% for 2004-2023). Female patients consistently exhibited higher survival (39.23% vs. 37.26% for males), with a pronounced age gradient 5-year RS declined from 45.71% in patients <45 years to 26.05% in those >74 years. The urban-rural survival gap narrowed, with urban areas showing 37.83% versus 37.25% in rural areas (2019-2023).

**Conclusion:**

This study provides the most current population-based long-term RS data for liver cancer in Taizhou, offering a critical benchmark for enhancing early detection and screening programs in eastern China.

## 1 Introduction

Liver cancer is the third leading cause of cancer-related mortality worldwide. According to the 2020 GLOBOCAN global burden estimates, approximately 905,677 new liver cancer cases and 830,180 deaths occur annually, with China accounting for 45.3% of global liver cancer incidence (1–3). From 2010 to 2021, global liver cancer incident cases and deaths increased by 26% and 25%, respectively (4). The number of new cases of global liver cancer per year is predicted to increase by 55.0% between 2020 and 2040. In addition, it is projected that the mortality rate of global liver cancer in 2040 will be 56.4% more than that in 2020 (5). The number of cancer cases and deaths, as well as crude incidence and mortality of cancer in China have increased gradually since 2000. In China, the latest estimated number of new cases and deaths due to liver cancer in 2022 was 370,000 and 320,000, ranking the 4th and 2nd for incidence and mortality rates among all malignant tumors, respectively (6). This staggering burden underscores the urgent need for region-specific preventive and therapeutic strategies.

Accurate evaluation of long-term survival outcomes through population-based cancer registries is pivotal for monitoring the evolving burden of liver cancer and guiding evidence-based public health interventions (7, 8). The 5-year RS rate, a cornerstone metric in oncological epidemiology, offers pivotal insights into the efficacy of early detection programs and therapeutic innovations, demanding continuous updates to align with real-world clinical advancements (9, 10). Traditional RS estimation methodologies, such as cohort and retrospective studies, require longitudinal follow-up data spanning a minimum of five years post-diagnosis, inherently introducing delays of 6–8 years when accounting for data aggregation and reporting timelines (11). In contrast, the period analysis estimates the long-term survival probability of newly diagnosed patients by utilizing the survival experience of all patients within a defined time window (12). It integrates contemporaneous data from overlapping intervals, enabling near real-time survival estimation and emerging as the gold standard for modern cancer surveillance in high-resource healthcare systems (13). Moreover, the period analysis includes all cases within the period of interest, which does not require follow-up data, calculating the actual survival estimates of newly diagnosed patients. It has been proved that period analysis provided more precise cancer survival estimates than cohort and complete methods and was widely used in the western (11). The application of this method not only provides clinicians and patients with more realistic prognostic information but also effectively addresses the time lag inherent in traditional cohort studies (14). First introduced by Brenner and Hakulinen in 2004, this methodology has been refined through model-based adaptations utilizing generalized linear models to predict survival trajectories for emerging patient cohorts (13). Despite robust validation across European and North American registries (15), fewer than 15% of Chinese registries adopt this analysis (16). At present, there is an extreme lack of research that systematically compares the long-term survival trends of specific cancers in China, such as liver cancer, with the advanced international levels using a unified period method model (17). In recent years, China has made significant breakthroughs in the targeted therapy and immune checkpoint inhibitors (ICIs) for liver cancer. The timely performance of the period analysis can accurately reflect the contemporary clinical treatment standards. This methodological stagnation hinders the generation of timely, policy-relevant survival metrics imperative for addressing regional disparities in cancer outcomes and optimizing resource allocation (18).

Furthermore, current population-based survival studies in China predominantly rely on retrospective cohort methods, which inherently obscure the immediate survival benefits derived from recent therapeutic advancements and comprehensive screening programs. A critical research gap persists regarding the lack of temporally refined, long-term survival data across varying socioeconomic regions, particularly in rapidly developing areas like eastern China. Because existing registries often lack granular clinical covariates, applying period analysis to provide real-time, macro-level survival surveillance is urgently needed. Consequently, this study holds substantial public health value for the local population, offering an essential, up-to-date benchmark to evaluate the efficacy of regional early detection initiatives and to guide targeted resource allocation.

Building on our prior validation of period analysis for Taizhou, eastern China (2014-2018) (19), this study extends the application of this methodology to four consecutive five-year intervals: 2019-2023, 2014-2023, 2009-2023, and 2004-2023. By leveraging updated registry data and model-based adaptations, we aim to generate temporally refined survival estimates that capture evolving therapeutic paradigms and demographic shifts in liver cancer epidemiology.

## 2 Methods

### 2.1 Data source

This study utilized data from liver cancer (ICD-10: C22.0) patients diagnosed between January 1, 2004, and December 31, 2023, across four regions in Taizhou, China: Luqiao District, Yuhuan City, Xianju County, and Wenling City. Initial data extraction identified 16,628 cases from local cancer registries. After excluding 2,329 non-cancer-related deaths, 1,087 cases lost to follow-up, and 1,167 cases with logical inconsistencies or missing variables (validated using IARCcrg Tools), and 13 patients aged ≤15 years to align with adult survival analysis protocols. The final analytical cohort comprised 12,032 eligible patients, stratified into four intervals: 2004-2023 (n = 12,032), 2009-2023 (n = 10,495), 2014-2023 (n = 7,472), and 2019-2023 (n = 3,536). The observed survival outcome was rigorously defined as the time interval from the date of initial liver cancer diagnosis to the date of death from any cause. To address temporal heterogeneity, the analysis incorporated left truncation (to exclude pre-2004 diagnoses) and right censoring (to account for patients lost to follow-up or alive at the study endpoint), thereby generating a dynamic survival cohort that reflects real-world clinical trajectories.

### 2.2 Statistical analysis

RS, a key metric for evaluating long-term cancer outcomes, was calculated as the ratio of observed survival in liver cancer patients to the expected survival of a matched general population (20). Following standardized RS methodology, we employed a population-based cohort design using cancer registry data to estimate 5-year RS rates. Specifically, the expected survival rates were derived from region-, gender-, and age-stratified general population life tables using the Ederer II method to account for competing mortality risks (21). This approach minimizes bias in survival estimation by aligning demographic characteristics between patient and reference populations, ensuring comparability across subgroups (22).

To capture the most recent trends in long-term survival outcomes, we implemented period analysis, a dynamic modeling approach for survival data, to evaluate liver cancer patients diagnosed between 2004 and 2023. Four age-stratified cohorts were constructed in accordance with global standard protocols:1) Cases diagnosed in 2004–2023 and alive as of December 31, 2023; 2) Cases diagnosed in 2009–2023 and alive during follow-up; 3) Cases diagnosed in 2014–2023 with active survival status; 4) Cases diagnosed in 2019–2023 under continuous observation. Follow-up was uniformly truncated at the end of 2023. The formula has been described in detail elsewhere (19, 23) (**Supplementary Material**).

Statistical analyses were performed using R (version 4.3.2). All survival rate estimates underwent age standardization based on the World Standard Population (WSP) age structure to ensure comparability across temporal periods and demographic subgroups. By integrating dynamic cohort construction and multivariate stratified analysis, this methodology effectively mitigates temporal bias inherent in conventional survival analyses, thereby generating time-sensitive evidence to inform liver cancer prognosis evaluation and clinical decision-making.

## 3 Results

### 3.1 Basic characteristics of liver cancer patients

The study cohort comprised 12,032 liver cancer patients diagnosed in Taizhou between 2004 and 2023 (**Table 1**). Male predominance persisted across all periods (77.7% of cases). A significant age-structural tr ansition was observed: the proportion of younger patients (<45 years) declined progressively from 7.7% (2004–2023) to 5.4% (2019-2023; annual reduction rate: 1.2%), while elderly patients (>74 years) increased from 17.1% to 18.2%. Notably, the 65-74-year subgroup surpassed the historically predominant 55-64-year cohort in 2019-2023 (28.1% vs. 30.1%), mirroring population aging trends in East Asia. Geographically, rural regions accounted for 87.1-88.6% of cases, consistently exceeding urban proportions (11.4-12.9%), reflecting systemic inequities in healthcare access and early detection capacity.

**Table 1:** Liver cancer diagnoses in Taizhou’s four counties and districts by period: 2019-2023, 2014-2023, 2009-2023, and 2004-2023.

Characteristics		Diagnosed interval			
		2019-2023 (%)	2014-2023 (%)	2009-2023 (%)	2004-2023 (%)
Total		3,534 (100)	7,472 (100)	10,495 (100)	12,032 (100)
Gender					
	Male	2748 (77.8)	5813 (76.8)	8155 (77.7)	9351 (77.7)
	Female	788 (22.2)	1659 (22.2)	2340 (22.3)	2681 (22.3)
Region					
	Urban	415 (11.7)	962 (12.9)	1356 (12.9)	1372 (11.4)
	Rural	3121 (88.3)	6510 (87.1)	9139 (87.1)	10660 (88.6)
Age at diagnosis (years)					
	<45	190 (5.4)	446 (6)	723 (6.9)	929 (7.7)
	45-54	644 (18.2)	1483 (19.9)	2,174 (20.7)	2496 (20.7)
	55-64	1065 (30.1)	2225 (29.8)	3127 (29.8)	3513 (29.2)
	65-74	995 (28.2)	1961 (26.3)	2652 (25.3)	3040 (25.3)
	>74	642 (18.1)	1357 (18.2)	1819 (17.3)	2054 (17.1)

### 3.2 Serial data on survival for overall and the stratification

To make a clear distinction, this section will report the age-standardized relative survival rate (ASRS). The ASRS is based on age-standardized according to the World Standard Population (WSP), specifically designed to eliminate differences in age structure and to assess the true survival trends among different calendar periods and regions. As shown in **Table 2**, the 5-year ASRS for liver cancer patients diagnosed during 2019–2023 reached 37.32%. A progressive decline in ASRS was observed with extended follow-up: 29.13% for 10-year RS (2014-2023), 23.00% for 15-year ASRS (2009-2023), and 5.56% for 20-year ASRS (2004-2023), indicating persistent challenges in improving long-term liver cancer prognosis.

**Table 2:** Age-standardized 5-, 10-, 15-, and 20-year relative survival rates of liver cancer patients in the four counties/districts of Taizhou. Using age weights based on the latest international standards for cancer survival comparisons, hereafter referred to as the ‘Global Standard’.

Age-standardized 5-, 10-, 15-, and 20-year relative rates of liver cancer patients in Taizhou		Age-standardized 5-year relative survival (2019-2023)		Age-standardized 10-year relative survival (2014-2023)			Age-standardized 15-year relative survival (2009-2023)		Age-standardized 20-year relative survival (2004-2023)	
		5-year RS (%)	Standard error	10-year RS (%)		Standard error	15-year RS (%)	Standard error	20-year RS (%)	Standard error
Total		37.32	0.97	29.13		0.94	23.00	1.30	5.56	3.04
Gender										
Male		37.26	1.10	27.07		1.05c	19.91	1.35	5.23	2.94
Female		39.23	2.11	37.69		2.14	32.92	2.09	`13.67	3.07
Age at diagnosis										
<45		45.71	3.60	33.16		2.64	23.09	2.71	15.74	3.23
45-54		43.44	2.16	30.19		1.75	19.17	1.95	13.16	3.65
55-64		40.62	1.74	25.19		1.48	18.23	2.07	5.09	4.27
65-74		32.66	1.78	24.43		1.80	17.49	3.34	4.49	4.23
>74		26.05	2.47	19.67		2.45	14.56	3.76	3.11	4.78
Region										
Rural		37.25	0.94	28.38		1.01	21.79	1.33	4.03	3.20
Urban		37.83	2.75	35.14		2.60	31.03	4.46	8.72	4.13

Gender-specific disparities revealed a consistent female survival advantage across all intervals. For instance, the 5-year ASRS was significantly higher in females (39.23%) than males (37.26%), with this gap widening to 8.44% by the 20-year follow-up (13.67% vs. 5.23%), suggesting potential biological or behavioral protective mechanisms unique to females. Urban populations maintained a modest survival advantage (5-year ASRS: 37.83% vs. 37.25% in rural areas), though this gap narrowed compared to earlier decades. Age-stratified analyses identified a pronounced negative gradient effect. The highest 5-year ASRS was observed in patients aged <45 years (45.71%), sharply declining to 26.05% in those >74 years. Similarly, 10-year ASRS decreased from 33.16% in the youngest cohort to 19.67% in the oldest, while 20-year ASRS declined from 15.74% to 3.11%.

### 3.3 Trends of 20-year RS during 2004-2023

**Figure 1:**
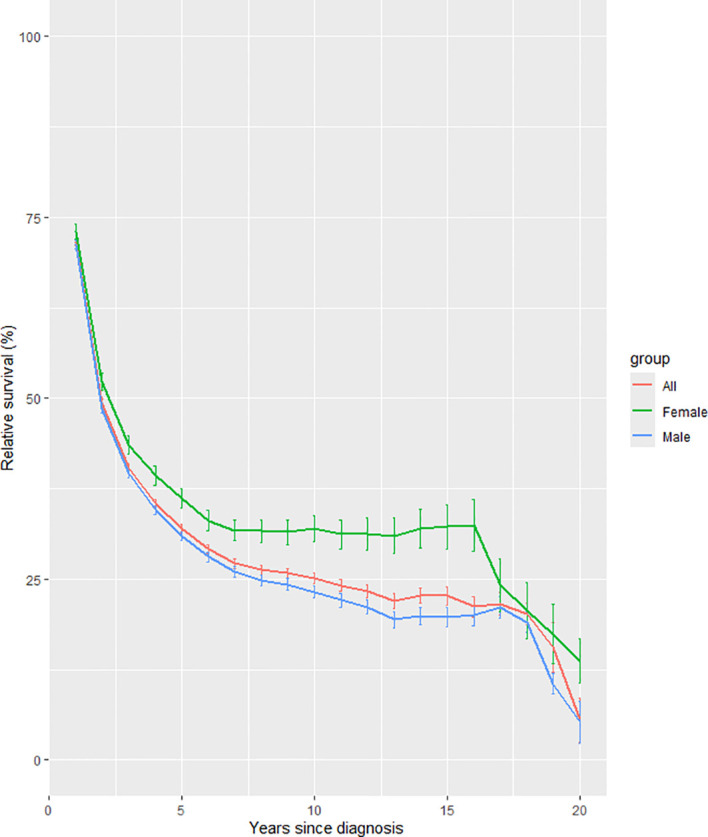
Trends of 20-year relative survival for liver cancer patients during 2004–2023 by gender.

**Figure 2:**
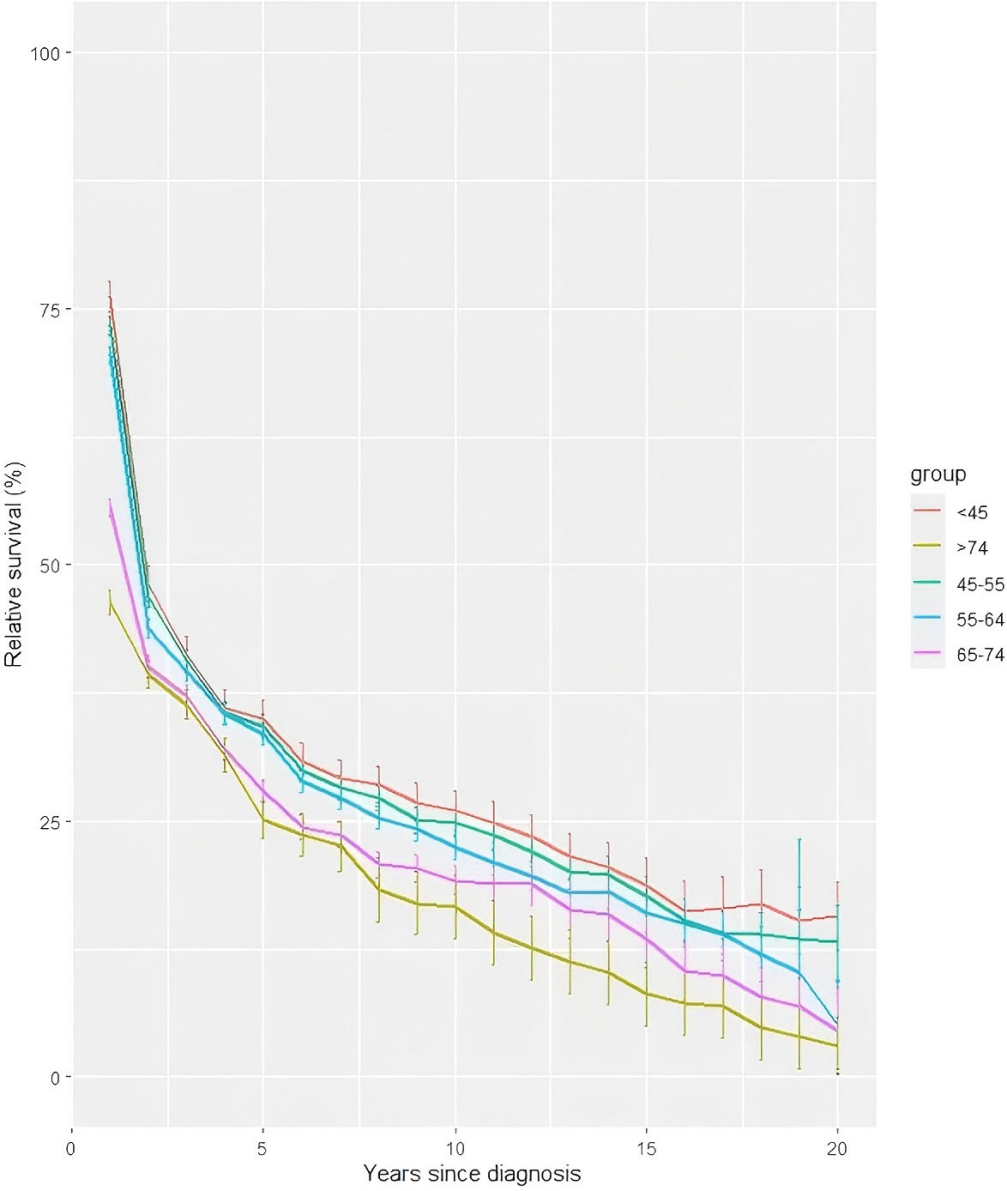
Trends of 20-year relative survival for liver cancer patients during 2004–2023 by age at diagnosis.

**Figure 3:**
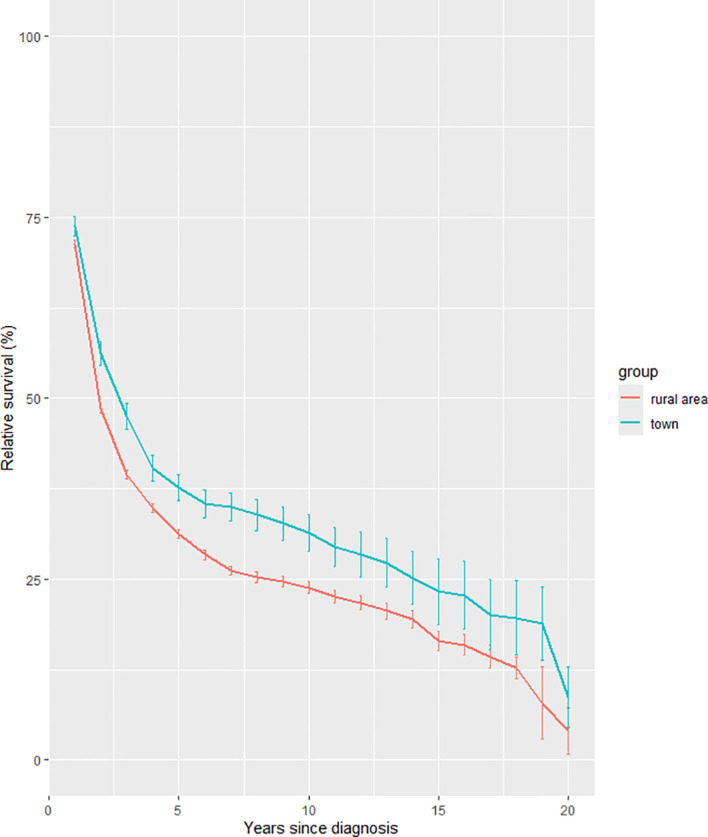
Trends of 20-year relative survival for liver cancer patients during 2004–2023 by region.

Longitudinal trend analyses stratified by gender (**Figure 1**), age at diagnosis (**Figure 2**), and geographic region (**Figure 3**) revealed persistent influences of these factors on 20-year RS among liver cancer patients from 2004 to 2023.

As shown in **Figure 1**, Female patients exhibited significantly higher ASRS (13.67%) compared to males (5.23%), with a nearly threefold difference. Female survival trajectories demonstrated gradual declines, stabilizing marginally after 15 years, whereas male survival rates declined significantly within the first decade post-diagnosis.

As shown in **Figure 2**, the youngest cohort (<45 years at diagnosis) achieved the highest 20-year ASRS (15.74%), contrasting sharply with an exceptionally high mortality rate in the oldest group (>74 years; 3.11%). Intermediate age groups (45-54, 55-64, and 65–74 years) displayed progressively lower survival rates (13.16%, 5.09%, and 4.49%, respectively). Notably, large standard errors in the oldest subgroup (>74 years) underscore challenges in obtaining precise estimates due to limited long-term follow-up in aging populations. Similarly, patients aged <45 years demonstrated delayed mortality attrition compared to elderly cohorts, whose survival rates dropped rapidly, likely due to comorbidities and limited treatment tolerance.

Urban-Rural Disparities (**Figure 3**): Urban populations sustained a survival advantage over rural counterparts (8.72% vs. 4.03%). Urban survival curves declined more gradually, particularly beyond 10 years, potentially reflecting enhanced access to specialized care, advanced therapies, or rehabilitation services. Conversely, rural populations experienced accelerated declines after 5 years, possibly linked to delayed detection, fragmented follow-up, or socioeconomic barriers.

## 4 Discussion

Building on our previous work, the present study represents the first application of period analysis in China to update the most current 5-year relative survival (RS) estimates for liver cancer patients in Taizhou, eastern China (2019-2023), and to comprehensively assess their long-term RS. Our findings address a critical gap in population-based survival surveillance, offering novel evidence to guide dynamic monitoring of liver cancer outcomes nationally. Our analysis revealed that the 5-year RS for patients diagnosed between 2019–2023 was 37.32%. Extended follow-up demonstrated a gradual decline in survival, with the 10-year RS (2014-2023) estimated at 29.13%, the 15-year RS (2009-2023) at 23.00%, and the 20-year RS (2004-2023) at 5.56%. Subgroup analyses further revealed that female patients consistently exhibited higher survival rates than their male counterparts with 5-year RS of 39.23% versus 37.26% for males, a disparity that widened to a threefold difference by the 20-year follow-up (13.67% vs. 5.23%). Similarly, patients residing in urban areas demonstrated significantly higher RS compared to those in rural regions (20-year RS: 8.72% vs. 4.03%), though the urban-rural gap narrowed in recent years (5-year RS: 37.83% vs. 37.25%). Furthermore, age-stratified analyses indicated that patients <45 years experienced a marked survival advantage, with the 5-year RS decreasing in an age-dependent manner from 45.71% in those <45 years to 26.05% in those >74 years.

Our analysis revealed that the 5-year RS for liver cancer patients in Taizhou during 2019–2023 was 37.32%, which is substantially higher than the 32.4% reported by Wang et al. for the 2014–2018 period (19). Both studies consistently demonstrate that female patients exhibit superior survival outcomes relative to their male counterparts, and age-stratified analyses further indicate that patients <45 years have more favorable outcomes than those >74 years. Our study not only updates the survival benchmarks established by Wang et al (19), but also expands their scope by incorporating extended follow-up intervals (10–20 years), thereby providing a more robust foundation for prognostic evaluation and optimization of long-term management strategies. Furthermore, the comparison of these studies underscores the critical importance of continuous survival monitoring, early detection, improved healthcare access, and optimized treatment protocols in enhancing liver cancer outcomes (24). The observed incremental improvement, from a 5-year RS of 32.4% to 37.32%, further offers compelling evidence of a favorable trend in future treatment prospects. It is noteworthy that a previous model-based period analysis by Wang et al. (2022) projected the 5-year RS for liver cancer in this region to reach 41.4% during 2019-2023. However, our actual observed estimate of 37.32% reveals that the previous projection was overly optimistic. This discrepancy can be attributed to several critical real-world factors. First, the 2019–2023 observational window heavily overlapped with the COVID-19 pandemic. The pandemic caused unprecedented disruptions to routine healthcare services, leading to delayed cancer screenings, postponed surgical interventions, and interrupted surveillance for patients with advanced liver disease (25). Then, the predictive model extrapolated future trends based on the rapid survival improvements observed between 2004 and 2018, which were largely driven by the initial dividends of the 2009 national healthcare reform and expanded medical insurance. In clinical reality, survival improvements tend to plateau once standard care access is saturated, making it statistically difficult to maintain a linear upward trajectory without disruptive therapeutic breakthroughs.

In comparison with similar international studies, our results demonstrate that the 5-year RS for liver cancer patients in Taizhou (37.32%, 2019-2023) is significantly higher than that reported in several high-income countries during 2010-2014 (5-30%) (26, 27). Specifically, liver cancer patients in China and several European countries, including the United States, the United Kingdom, Australia, Brazil, Argentina, and Italy, exhibited 5-year RS values ranging from approximately 10% to 19%, whereas regions such as South Korea, Singapore, and Belgium reported rates between 20% and 29%, with Japan achieving the highest rate at 30% (28, 29). In contrast, several Asia-Pacific countries, including Thailand and Russia, reported 5-year RS values below 10% (15). These discrepancies may be attributed not only to variations in the pathological stage at diagnosis and the timing of interventional treatments but also to differences in tumor histology, the recurrence of advanced liver disease or cancer, and patients’ baseline health status.

When compared to other domestic studies, Liver cancer remains the second leading cause of cancer-related mortality in Taizhou. Population-based cancer surveillance data from 2018 to 2023 indicate a five-year relative survival (RS) rate of 37.32% for liver cancer patients in the region, substantially higher than the 16.8% reported for Zhejiang Province during 2005-2010 (30) and exceeding the provincial estimate of 15.6% published in 2023 (31). These disparities can be attributed to several interrelated factors. First, sustained regional economic development, near-universal coverage of basic medical insurance, an efficient disease surveillance system, and the systematic implementation of large-scale early detection programs have collectively improved early diagnosis and timely treatment of liver cancer (32). Second, recent advances in therapeutic strategies-particularly the integration of surgical resection, targeted therapy, and immunotherapy into multimodal treatment frameworks-have significantly enhanced clinical outcomes and contributed to steady gains in survival rates (33). Furthermore, Taizhou’s five-year relative survival rate is notably higher than the national average of 12.1% reported for 2012-2015 (34). This national estimate was derived from aggregated data across 17 population-based cancer registries, which included only patients diagnosed by the end of 2013 with follow-up completed by 2015. As a result, the survival estimates are restricted to cases diagnosed up to 2013, with survival beyond this period based on model-based projections that may introduce estimation bias. In contrast, the present study draws on data from a representative cancer registry in eastern China, featuring rigorous data quality control, comprehensive case ascertainment, and integration within a well-developed healthcare system. This enables a more accurate and up-to-date assessment of current survival outcomes among patients with liver cancer. Moreover, promoting this gold-standard period analysis within the “Healthy China 2030” framework is urgently required to dynamically monitor the evolving cancer burden and rigorously validate the mandated 46.6% 5-year survival target.

Urban patients exhibit significantly higher RS compared to rural counterparts. This disparity is likely attributable to several interrelated factors. Firstly, substantial differences in the distribution of healthcare resources are evident; urban regions benefit from state-of-the-art medical equipment and comprehensive early screening initiatives, which facilitate timely disease detection and the delivery of precise treatments (35, 36). Secondly, lifestyle factors play a crucial role, as urban residents tend to adopt healthier dietary practices and engage in regular health management (37). Furthermore, higher education levels and increased health awareness among urban populations promote earlier symptom recognition and proactive healthcare-seeking behavior (38). Lastly, the presence of a well-established health insurance system, coupled with higher economic status in urban areas, ensures timely treatment and enhanced access to medical services (39).

Furthermore, the observed improvement in the 5-year RS for liver cancer patients is strongly linked to early diagnosis and timely treatment (40). Previous studies have reported that the 5-year survival rate can reach 83.9% for stage I liver cancer, 75.8% for stage II, 59.6% for stage III, and only 39.9% for stage IV (41). Advances in treatment strategies, improvements in perioperative management, and the integration of a multidisciplinary team (MDT) approach may further enhance long-term survival outcomes (42). Notably, Japan currently leads the world in 5-year RS, a success largely attributable to the widespread implementation of interventional treatment techniques (43, 44). Additionally, the utilization of minimally invasive procedures, such as laparoscopic surgery and the Da Vinci robotic system, has significantly reduced surgical trauma and steadily improved treatment efficacy, thereby benefiting liver cancer patients across all stages, including elderly individuals (45).

Our study demonstrates both considerable strengths and notable limitations. On the one hand, this study represents the first systematic evaluation of the latest 5-year relative survival (RS) rate for liver cancer patients in Taizhou using period analysis, and it additionally provides long-term RS estimates for 10-, 15-, and 20-years. Furthermore, the use of population standardization techniques enhances the precision and reliability of our findings. On the other hand, the study is limited by the absence of data regarding tumor stage, histological subtype, and treatment details, thereby constraining a more in-depth exploration of the underlying mechanisms. Moreover, considering that the healthcare infrastructure in Taizhou is relatively advanced, the findings may not be fully generalizable to national trends.

## 5 Conclusion

In conclusion, our study demonstrates that Taizhou serves as a paradigm for liver cancer management in China, with survival outcomes nearing international benchmarks. The observed improvement in the 5-year relative survival rate, together with the introduction of innovative long-term survival metrics, substantiates the efficacy of early screening initiatives. Nonetheless, persistent disparities in survival between rural and urban populations, as well as across different age groups, underscore the necessity for targeted interventions, such as deploying mobile screening units in rural areas and establishing dedicated geriatric oncology protocols. Future investigations should incorporate molecular and socioeconomic data to refine risk stratification, thereby ensuring that improvements in survival are equitably realized across all demographic subgroups.

## Data Availability

The original contributions presented in the study are included in the article/**Supplementary Material**. Further inquiries can be directed to the corresponding authors.
