# Supplementary material for: Using period analysis to timely provide serial data on long-term survival for liver cancer patients from China
Source: Front Oncol. 2026 Apr 14;16:1801386. doi: 10.3389/fonc.2026.1801386 (PMC13120958; doi:10.3389/fonc.2026.1801386)
Supplement: Supplementary file 1 [file DataSheet1.docx]

**Supplementary Material**

**Using Period Analysis to Timely Provide Serial Data on Long-term Survival for Liver Cancer Patients from China**

Pengtao Chen^1, 2, 3#^, Wei Gu^1, 2, 3#^, Xin Bing^2, 3, 4#^, Liangyou Wang^5^, Yongran Cheng^6^, Xukai Chen^2, 3^, Dinghu Zhang^1, 3, 7*^, Tianhui Chen^1, 2, 3*^

^1^Postgraduate training base Alliance of Wenzhou Medical University (Zhejiang Cancer Hospital), Wenzhou, 325000, China

^2^Department of Cancer Prevention, Zhejiang Cancer Hospital, Hangzhou 310022, China

^3^Hangzhou Institute of Medicine (HIM), Chinese Academy of Sciences, Hangzhou 310018, China

^4^Wuwei Maternal and Child Health Hospital, Wuwei 733000, China

^5^Department of Non-communicable Chronic Disease Control and Prevention, Taizhou Center for Disease Control and Prevention, Taizhou 318000, China. School of Public Health, Hangzhou Medical College, Hangzhou 310013, China

^6^School of Public Health, Hangzhou Medical College, Hangzhou 310013, China

^7^Department of Interventional Radiology, Zhejiang Cancer Hospital, Hangzhou, 310022, China

# These authors contributed equally to this work.

*** Correspondence:**Tianhui Chen, Postgraduate training base Alliance of Wenzhou Medical University (Zhejiang Cancer Hospital), Wenzhou, 325000, China; Department of Cancer Prevention, Zhejiang Cancer Hospital, Hangzhou 310022, China; Hangzhou Institute of Medicine (HIM), Chinese Academy of Sciences, Hangzhou 310018, China;
[chenth@zjcc.org.cn](mailto:chenth@zjcc.org.cn)

Telephone: 15267055007

**Period analysis was used to calculate 5-, 10-, 15-, 20-year RS.**

Following data standardization, the life-table model was employed to calculate annual conditional survival probabilities stratified by follow-up duration. The conditional survival probability for the i-th year (S_i_) was derived using the formula:

$$\begin{aligned} S_{i}=1-\frac{d_{i}}{n_{i}- {c_{i}}/2}\#\left( 1 \right) \end{aligned}$$

where n_i_ denotes the number of patients at risk at the beginning of the i-th year, di represents the observed deaths during year i, and c_i_ corresponds to the censored cases within the same interval.

The cumulative k-year observed survival rate ($\bar{S_{k}}$) was calculated as the product of annual conditional survival probabilities:

$$\begin{aligned} \bar{S_{k}}=\prod_{i=1}^{k} S_{i}\#\left( 2 \right) \end{aligned}$$

The k-year RS rate (R_k_) was then defined as the ratio of the observed survival rate ($\bar{S_{k}}$) to the expected survival rate (Sk^∗^) of the matched general population:

$$\begin{aligned} R_{i}= \frac{\bar{S_{k}}}{S_{k}^{*}}\#\left( 3 \right) \end{aligned}$$

When k was assigned values of 5, 10, 15, and 20, RS estimates were generated for corresponding follow-up intervals. RS point estimates and their standard errors (SE) were computed via the Greenwood formula to quantify uncertainty in survival estimates.
